# COX-2 Inhibitors and Gastric Cancer

**DOI:** 10.1155/2014/132320

**Published:** 2014-10-12

**Authors:** Zhen Wang, Jun-qiang Chen, Jin-lu Liu

**Affiliations:** Department of Gastrointestinal Surgery, The First Affiliated Hospital of Guangxi Medical University, 6 Shuangyong Road, Nanning, Guangxi Zhuang Autonomous Region 530021, China

## Abstract

The evidence that cyclooxygenase-2 (COX-2) is upregulated and plays an important role in carcinogenesis of gastric cancer has triggered the topic of COX-2 inhibitors as chemopreventive agents for gastric cancer. Studies find that COX-2 inhibitors are associated not only with chemoprophylactic effects, but also with chemotherapeutic potentials in gastric cancer. Both COX-dependent and COX-independent pathways have a role in the anticancer efficiency of COX-2 inhibitors. However, enthusiasm is thwarted by the potential toxicity, that is, gastrointestinal toxicity of nonselective COX-2 inhibitors and cardiovascular risk of selective COX-2 inhibitors. Therefore, more studies are needed to develop new targeted antitumor agents (such as prostaglandin E receptor antagonist) and to define fundamental questions such as optimal treatment regimens, integration of cotherapy, and careful selection of candidates.

## 1. Introduction

Gastric cancer (GC) is the fourth most common cancer and the second leading cause of cancer deaths worldwide [1]. Notwithstanding the global declining incidence of GC (especially in the western world), mortality is still rising in Asian countries. The prognosis of GC is improved significantly because of early diagnosis and treatment; however, the 5-year survival rate is less than 20% in individuals with advanced disease [2]. Low rate of radical gastrectomy and the intrinsic resistance to radio- and chemotherapy of GC may account for these dismal statistics. Therefore, primary prevention is likely to be the most effective means of reducing the incidence and mortality from this disease.

Although the etiology of GC is not fully understood, gastric carcinogenesis is known as a multistep and multifactorial process, such as chronic inflammation, to malignant lesions [3]. The process often spans over a long time, which provides a window of opportunities for effective interventions and prevention. Clinical observations have found that the use of nonsteroidal anti-inflammatory drugs (NSAIDs) is associated with reduced incidence of GC [4]. The main target of NSAIDs is the cyclooxygenase (COX) enzyme which catalyses the conversion of arachidonic acid to prostaglandins (PG). Two isoforms of COX are known: COX-1 and COX-2. COX-1 is constitutively expressed in many tissues, while COX-2, normally absent or expressed at very low levels in most tissues, is responsible for inflammatory reactions and tumor developments [5]. Several studies have reported that induction of COX-2 is associated with inhibition of apoptosis, increasing in angiogenesis and metastatic potential. Inhibition of COX-2 results in growth inhibition of GC* in vivo* and* in vitro* [6, 7]. More recently, studies show that COX-2 expression is upregulated in GC as well as in precancerous lesions and in* Helicobacter pylori*- (Hp-) induced inflammation, suggesting that COX-2 may play an important role in gastric carcinogenesis [8, 9]. Therefore, the relatively early role of COX-2 in gastric carcinogenesis makes it an attractive target for cancer chemoprevention. The chemoprevention of COX-2 inhibitors on GC seems rational, whereas the data of a series of clinical studies remain inconclusive [10]. Further understanding of the pharmacology and pharmacokinetics of COX-2 inhibitors may be helpful to make better strategies both for GC chemoprevention and for avoiding adverse reactions.

In this review, we search related studies as well as epidemiological observations in PubMed to provide a comprehensive examination of the COX-2 inhibitors in the clinical use of GC.

## 2. The Expression of COX-2 in Gastric Cancer

Normal gastric mucosa scarcely expresses COX-2, but COX-2 expression and PGE2 levels are upregulated through the multistep process of gastric carcinogenesis [9]. Since Ristimäki et al. described an elevated expression of COX-2 in GC for the first time [8], numerous studies have evaluated the relationship between COX-2 expression and gastric carcinogenesis. Sun et al. found that the positive rates of COX-2 by immunohistochemistry in superficial gastritis, gastric atrophy, intestinal metaplasia, dysplasia, and cancer were 10.0%, 35.7%, 37.8%, 41.7%, and 69.5%, respectively [11]. Thus, it seems likely that COX-2 plays a role in early gastric carcinogenesis, despite the fact that the precise mechanisms leading to the elevated expression of COX-2 are still not fully clarified. Nevertheless, evidences suggest that proinflammatory cytokines, gastrin, mitogen, and growth factors could be involved in the process [12, 13]. Interestingly, previous studies mainly focused on the upregulated COX-2 mRNA and the overexpression of COX-2 protein; copy number gain of the COX-2 gene at the DNA level has not been reported in GC [14].

In addition, COX-2 expression is associated with the clinical outcome of GC. The COX-2 expression is more frequent in intestinal type than in diffuse type GC and more frequent in proximal location than in distal location [15, 16]. Meanwhile, studies report that the level of COX-2 expression is correlated with tumor size, depth of invasion, lymph node metastasis, lymphatic invasion, clinical stage, and prognosis [13, 17]. However, the association remains controversial, since some studies show no such link [18]. Shi et al. find that COX-2 expression is an independent prognostic factor in patients with GC [19], whereas some researchers find that the indication effect of COX-2 expression in the prognosis of GC is affected by the tumor stage [20]. Therefore, whether COX-2 could identify those patients with nonadvanced, operable, but potentially aggressive cancer is worthy of being studied.

## 3. COX-2 Expression and* Helicobacter pylori* Infection

*Helicobacter pylori* (Hp) has been regarded as one of definite carcinogens in GC according to recent epidemiologic evidences. Indeed, the colonization of gastric mucosa with Hp causes a chronic inflammatory reaction with increased generation of reactive oxygen species and production of proinflammatory cytokines [21]. Chronic atrophic gastritis caused by Hp activates synthesis of growth factors and cytokines leading to elevated COX-2 expression [22]. Studies* in vitro* find that Hp correlates with an upregulation of the expression of COX-2 mRNA/protein and PGE2 in GC cell lines [23]. Additionally, studies in rat model find that gastric epithelial cells treated with Hp water extract (only containing bacterial proteins but not bacterial cells) leads to an increase in COX-2 and PGE2 levels that peaked 24 h after treatment and declined at 48 h [24]. These suggest that Hp plays an important role in induction of COX-2 synthesis during chronic gastritis which is a precancerous condition for GC. Therefore, inhibiting the expression of COX-2 combined with the eradication of Hp may be efficient in prevention of GC.

## 4. COX-2 Inhibitors in Prevention of Gastric Cancer

Chemoprevention is referred to the prevention of cancer using specific agents to suppress or reverse the carcinogenic process. Chemoprevention has been developed in the absence of other validated methods. In order to reduce the incidence of cancer effectively, chemopreventive agents must fulfill several criteria. First and most importantly, they should have acceptable side effects because toxic effects will affect mortality and complications. Second, the agent must be cost-effective because patients will not be able to undertake what will be many years of lengthy expenditure for “invisible effects.” Lastly, they need to be acceptable to patients taking them and their mechanism should be clear so they remain motivated. In spite of the huge list of potential chemopreventive agents, there are no agents licensed for chemoprevention in adults until now. NSAIDs, including aspirin and COX-2 agents in prevention of GC, gain the most recent interest [25].

Epidemiological studies clearly indicate that prolonged NSAID use is associated with a reduced risk of cancer; meanwhile,* in vitro* and* in vivo* studies show that some NSAIDs are effective in the treatment and prevention of GC. One of the oldest agents that has recently been known to have cancer chemopreventive effects is aspirin, which has been used in clinical practice since the 19th century [26]. Several case-control studies have examined the potential preventative effect of aspirin or NSAID use on GC. In a study conducted in Los Angeles County, the preventative effect of aspirin or NSAID use on GC was evaluated by including cases with cardia cancer, noncardia cancer, and controls. Aspirin or NSAID use in excess of 5 years was associated with reduced odds of distal GC but not cardia cancer [27]. There appeared to be a dose-response effect, with the greatest reduction in odds observed in those who took at least a pill per day. Prospective cohort studies of this issue have also been performed. In a cohort of almost 200,000 subjects, a significant trend for a decreasing risk of distal GC with increasing use of aspirin was observed. However, there was no association between either aspirin or NSAID use and cardia cancer [28]. To provide an up-to-date quantification of this association, a meta-analysis including seven case-control and six cohort studies on aspirin and GC was conducted [29]. As a result, regular aspirin use was associated with a 33% reduction in the relative risk of GC. Nevertheless, dose-risk and duration-risk relationships are not analyzed in the study. To avoid this problem, Ye et al. carried out a dose-response meta-analysis to evaluate the threshold effect between aspirin intake and the risk of GC, and they found that long-term (≧4 years) and low-frequency (1–4.5 times per week) aspirin use was associated with a statistically significant, dose-dependent reduction in the risk of GC [30].

Generally, observational studies and meta-analysis have their intrinsic shortages, such as uncertain causality and confounding factors. What we need are rigorously designed RCTs to evaluate the effect of aspirin or NSAIDs on the incidence of GC in high-risk populations. To date, very few reliable data can be referenced. However, based on individual patient data from RCTs of aspirin, two meta-analyses published recently found that regular aspirin use appeared to be protective against GC, which were highly correlated with those in observational studies [31, 32]. These data suggest that primary prevention trials of aspirin in populations at high-risk of GC are warranted.

Although aspirin and NSAIDs have significantly protective effect against GC, we must notice their side effects in clinics, such as gastrointestinal bleeding, hemorrhagic stroke, reduction of the renal blood flow, dysfunction of platelets, and, rarely, allergic reactions. It is reported that aspirin increases the gastrointestinal bleeding rate 2- to 4-fold, especially in patients over 70 years of age [33]. That is why aspirin or NSAIDs cannot be given to everyone. The gastrointestinal toxicity of aspirin and NSAIDs is mediated by the inhibition of COX-1 enzyme. To avoid those side effects of nonselective NSAIDs, the development of selective COX-2 inhibitors was gradually started after the discovery of COX-2 in tumorigenesis since the early 1990s. Selective COX-2 inhibitors (COXIBs) help to alleviate these complications by selectively limiting COX-2 activity, while sparing COX-1 can mediate cytoprotective effects on the gastrointestinal mucosa in terms of increasing mucosal blood flow, reducing gastric acid secretion and stimulating the release of viscous mucus [10].

Several* in vitro* studies have analyzed the effect of the selective COX-2 inhibitors on GC cell lines focusing on cell proliferation and apoptosis. The COX-2-specific inhibitor NS-398 induced apoptosis and suppressed cell proliferation in MKN45 cell lines, which abundantly express COX-2 [34]. Moreover,* in vivo* experimental studies evaluated the effect of selective COX-2 inhibitors in GC by using drug-induced model. When the selective COX-2 inhibitor nimesulide was administered long-term, gastric tumorigenesis was significantly attenuated in N-methyl-N-nitrosourea-treated mice and apoptosis was increased in these tumors [35]. Recently, the chemopreventive effect of celecoxib, a COX-2-selective inhibitor, was also confirmed in GC patients [36].

Although current evidence showed the chemopreventive effect of COX-2 inhibitors in GC, RCTs are needed to confirm the reliability of such sources. Additionally, fundamental questions such as safety, mechanisms of actions, and optimal treatment regimens need to be defined.

## 5. COX-2 Inhibitors in Treatment of Gastric Cancer

Previous study mainly focused on the chemoprophylactic effect of NSAIDs (including selective COX-2 inhibitors) in GC; however, the chemotherapeutic potential of NSAIDs in GC was uncertain. Recently,* in vitro* studies suggested that celecoxib could reverse multidrug resistance in human GC by downregulation of the expression and activity of P-glycoprotein [37]. In addition, Zhu et al. found that rofecoxib (a selective COX-2 inhibitor) played a chemotherapeutic sensitizer role in various anticancer agents on the BGC-823 gastric cancer cell line and explained the mechanism by using its ability to reverse the intrinsic MRP1 (multidrug resistance-associated protein 1) and GST-p (glutathione S-transferase-p)* in vitro*, which were caused by downregulation of the expression and activity of P-glycoprotein [38]. By establishing nude mice animal model, Tendo et al. found that combining S-1 (a novel 5-fluorouracil derivative providing high clinical response rates without severe adverse effects) and COX-2 inhibitor administration obtains a synergistic inhibitory-effect on the peritoneal metastasis of scirrhous GC [39]. These showed that COX-2 inhibitor alone or combined with other anticancer agents could be used in the treatment of GC.

Nevertheless, Chen et al. found that celecoxib combined with cisplatin did not elicit greater antitumor activity than cisplatin or celecoxib monotherapy* in vivo* in a gastric xenograft model and concluded that treatment strategies with celecoxib in combination with cisplatin should act cautiously [40]. By further investigation, they considered that the antagonizing effect of celecoxib on the cytotoxicity of cisplatin resulted from its special chemical structures (decreasing intracellular cisplatin accumulation and the extent of cisplatin-DNA adduct formation) rather than its COX-2 inhibitory activity. Therefore, we think that COX-2 inhibitors with different chemical structures may exhibit different effects when combined with other anticancer agents, and treatment strategies with COX-2 inhibitors in combination with other anticancer agents should act cautiously in clinics. In addition, previous reports are mainly* in vitro* or* in vivo* studies, and human trials are very few; hence more studies are needed to confirm the chemotherapeutic effect of COX-2 inhibitors in GC before wide usage in clinics.

## 6. Possible Mechanisms in Chemoprevention of Gastric Cancer

The antitumor effect of NSAIDs is thought to be caused by COX-2 inhibition and the consequential reduction of prostaglandin (PG) synthesis, which is called COX-2-dependent anticancer mechanism. COX is a rate-limiting enzyme in the conversion of arachidonic acid to PGH2, which is subsequently converted to other PGs and thromboxane A2 by specific PG- and thromboxane-synthases. Among different types of PGs catalyzed by COX-2, PGE2 reaches high levels in tumor tissues and plays a central role in carcinogenesis by regulating a number of cellular behaviors related to the tumorigenesis of stomach, including premalignant lesion formation, cancer-associated angiogenesis, and the invasion and metastasis of cancer [41]. PGE2 can cause the expansion of tumor mass by stimulating proliferation and suppressing apoptosis of GC cells and promote cancer-associated angiogenesis by supplying nutrients and oxygen to the tumor and providing a route for metastasis [42]. Moreover, PGE2 has also been implicated in enhancing GC cell invasiveness, facilitating escape from immune surveillance, and inducing resistance to chemotherapeutic agents [42]. Studies found that both cell proliferation and PGE2 levels were significantly reduced following COX-2 inhibition; additionally, both COX-2 inhibitors and PGE2 receptors (EP2) antagonists could inhibit angiogenesis and tumor invasion in GC cells [43]. These observations implicated that reduction of PGE2 was considered as the important anticancer pathway of COX-2 inhibitors. Besides PGE2, other prostanoids such as PGI2 were also involved in carcinogenesis through their direct effects on cancer cells, which are associated with the anticancer effects of COX-2 inhibitors [10].

However, accumulated evidences suggest that the tumor-inhibitory efficacy of nonselective NSAIDs or selective COX-2 inhibitors is not necessarily related to their COX-inhibitory ability. COX-2 inhibitors can inhibit proliferation and induce cell apoptosis in cells that do not express COX [44], and NSAID derivates that lack the ability to inhibit COX (sulindac sulfone) can inhibit gastrointestinal tumor growth* in vivo* and* in vitro* [45]. These suggest other targets may play a role in the antitumor effect of COX-2 inhibitors, which is called COX-2-independent anticancer mechanism. Potential mechanisms involve the induction of apoptosis by inhibiting NF-*κ*B signaling pathway [46], facilitating P53-induced cell death [47], upregulating the NAG-1 pathway, or increasing 15-LOX-1 activity [48, 49]. Moreover, COX-2 inhibitors can exert antiproliferation effect by suppressing telomerase activity [50]. Besides, some researchers found that selective COX-2 inhibitor (celecoxib) could exert antitumor effect by cell cycle arrest [10].

## 7. Side Effects and Cost-Effectiveness Analysis 

Extensive evidences based on clinical reports indicate that regular intake of various NSAIDs reduces the risk of GC, whereas various adverse events have been reported due to long-term use of nonselective and selective NSAIDs. As we know, the toxicity of NSAIDs is a consequence of the inhibition of the COX enzymes.

Generally, gastrointestinal toxicity is the most common nonselective NSAID-induced adverse events, which is caused by the inhibition of COX-1 enzyme. It is reported that NSAIDs are associated with an increased rate of gastrointestinal bleeding (2- to 4-fold) and a 15%–30% prevalence of ulcers in the stomach/duodenum, especially in patients over 70 years of age [10, 33]. Approximately 1% of patients who regularly take NSAIDs for 3 to 6 months suffered from symptomatic ulcers or ulcer related complications. The incidence will increase to 2%–4% if patients take them for 1 year, and 80% of patients may have no preceding symptoms [10]. Of course, the risk of gastrointestinal events is dependent on the patient characteristics at baseline (such as* H. pylori* infection and previous ulcer history), the dose, and the protection conferred by cotherapy. When nonselective NSAIDs are given with a PPI, especially after* Helicobacter pylori* eradication, the risk of gastrointestinal events after NSAID use is dramatically decreased [51]. AspECT, a randomized trial, to assess potentially synergistic agents (aspirin and esomeprazole) dealing with both potential anticancer effects and cardiac protective effects, found that the incidence of serious gastrointestinal side effects is very low [52]. The optimal dose of NSAIDs for GC prevention remains unknown. Large, population-based studies find that a larger dose may be given for chemoprevention compared with cardioprotection [25]. These larger doses would increase the risk of gastrointestinal toxicity; however, would combining PPI with NSAIDs be effective? This remains a question to be answered.

To avoid the gastrointestinal toxicity of nonselective NSAIDs, selective COX-2 inhibitors were gradually developed. However, these COX-2 inhibitors have recently come under intense scrutiny because of clinical reports linking COXIBs to increased risk of serious cardiovascular harm, such as unstable angina, myocardial infarction, cardiac thrombus, ischemic stroke, sudden or unexplained death, resuscitated cardiac arrest, and transient ischemic attacks [53]. In September 2004, rofecoxib was withdrawn from the US market because of a possible increased risk of serious cardiovascular harm. Celecoxib was allowed to remain in the market place but with a black box warning indicating a risk of adverse cardiovascular events. Nevertheless, Feng et al. found that celecoxib, compared with placebos, did not increase the risk of cardiovascular events in the treatment of GC [54]. The exact mechanisms for the increase in cardiovascular risk of COX-2 inhibitors remain somewhat elusive. Selective COX-2 inhibitors may suppress vascular synthesis of prostaglandins without affecting the production of platelet-derived thromboxane A2. The imbalance may promote thrombosis and increase the risk of cardiovascular events [36]. In order to gain the benefit and avoid the cardiovascular events from clinical chemoprevention of GC, optimal regime of selective COX-2 inhibitors by adjustment of dosage and duration and careful selection of high risk candidates is needed.

Long-term usage of COX-2 inhibitors is a high cost; therefore, cost-effectiveness analysis should be carried out to optimize the allocation of resources. Studies found that aspirin as chemoprevention against esophageal adenocarcinoma is cost-effective, assuming a risk reduction of 50% and 0.5% per year progression rate from Barrett's esophagus to cancer [25], whereas epidemiological data indicate that cumulative probability of developing a lesion from birth to 80 years of age is less than 4%. In the general population, over 95% of people treated prophylactically with COX-2 inhibitors will not benefit [55]. Therefore, chemoprevention with COX-2 inhibitors may be a worthwhile goal only in those subjects with a high risk of GC.

## 8. Conclusions and Future Directions

GC remains a major health concern. COX-2 is upregulated in GC and in its precursor lesions, and it provides valuable clinical information as a prognostic factor. Widespread and long-term use of NSAIDs has been advocated for GC chemoprevention in the healthy population. Nevertheless, enthusiasm has been thwarted by their gastrointestinal toxicity. In order to minimize the gastrointestinal side effects, selective COX-2 is developed and put into use in the chemoprevention of GC. However, we must keep in mind that COXIBs have also been linked to serious cardiovascular events. Therefore, to circumvent the toxic effects of NSAIDs and COX-2-selective inhibitors, combining profound acid-suppressing drugs with NSAIDs and development of new targeted antitumor agents (such as prostaglandin E receptor antagonist) would be expected to address these concerns.

Studies found that COX-2 inhibitors may inhibit the development of GC through inhibition of COX-2. Nevertheless, COX-2-independent mechanisms also contribute significantly to the chemopreventive effect. Therefore, the mechanisms of the antitumoural action of COX-2 inhibitors still remain to be defined. Previous studies mainly focus on the chemoprophylactic effect of COX-2 inhibitors in GC; however, their chemotherapeutic potential in GC is still to be confirmed. In addition, cost-effectiveness analysis should be conducted to optimize the allocation of resources. Moreover, prospective clinical trials are needed to define fundamental questions such as optimal treatment regimens, the age at which to initiate therapy, the optimum treatment duration, and the subpopulations for which the benefits of chemoprevention outweigh the risks of adverse side effects.
